# Solitary Fibrous Tumor of the Peritoneal Cavity and Greater Omentum: Case Report and Review of the Literature

**DOI:** 10.15388/Amed.2023.30.1.7

**Published:** 2023-03-06

**Authors:** Neda Gendvilaitė, Dmitrij Šeinin, Laurynas Beržanskas, Tomas Poškus

**Affiliations:** Faculty of Medicine, Vilnius University, M. K. Čiurlionio 21, LT-03101, Vilnius, Lithuania; National Center of Pathology, Affiliate of Vilnius University Hospital Santaros Klinikos, P. Baublio 5, LT-08406 Vilnius, Lithuania; National Center of Pathology, Affiliate of Vilnius University Hospital Santaros Klinikos, P. Baublio 5, LT-08406 Vilnius, Lithuania; Faculty of Medicine, Vilnius University, M. K. Čiurlionio 21, LT-03101, Vilnius, Lithuania

**Keywords:** Solitary fibrous tumor, greater omentum, soft tissue tumor

## Abstract

**Background:**

The solitary fibrous tumor is a rare soft tissue mesenchymal tumor which typically arises from the pleura but may be found anywhere in the body. Abdominal localizations are very rare. The solitary fibrous tumor is classified into two forms, pleural and extrapleural, and morphologically they resemble each other. The diagnostics of the tumor is usually accidental because usually there are no clinical symptoms. The main treatment of the solitary fibrous tumor is the surgical removal of the tumor while radiotherapy treatment and chemotherapy outcomes remain unclear due to the rarity of the tumor and lack of data.

**Case presentation:**

We report the case of the 32-year-old man who was diagnosed with the solitary fibrous tumor of the peritoneal cavity. Laparotomy was performed. A grayish-white, stiff, coarse-grained tumor about 11 cm in diameter of the greater omentum was found and radical omentectomy with tumor removal were performed. Postoperative course was uneventful and the patient is well with no signs of recurrence on the CT scan of the chest and abdomen and MRI of the pelvis at 6 months after surgery.

**Conclusions:**

The solitary fibrous tumor is a rare condition. It is a borderline-malignant tumor but may cause serious complications if not treated. Due to the absence of clinical symptoms, the tumor is usually detected accidentally. The radical surgical removal of the tumor is the most optimal treatment.

## Santrauka.

**Apžvalga:**

Solitarinis fibrozinis navikas yra retas minkštųjų audinių mezenchiminis navikas, jis dažniausiai kyla iš pleuros, bet gali būti aptinkamas bet kurioje kūno vietoje. Pilvo ertmės navikai yra gana reti. Solitarinis fibrozinis navikas klasifikuojamas į pleuros ir ekstrapleurinius navikus, tačiau morfologiškai jie yra panašūs. Navikas dažniausiai aptinkamas atsitiktinai kito nesusijusio tyrimo metu. Taip nutinka, nes navikas dažniausiai nesukelia jokių klinikinių simptomų. Pagrindinis solitarinio fibrozinio naviko gydymas yra chirurginis jo pašalinimas, nes radioterapinio gydymo ir chemoterapijos rezultatai nėra aiškūs dėl naviko retumo ir duomenų trūkumo.

**Klinikinis atvejis:**

Pristatomas 32 metų paciento, kuriam buvo diagnozuotas pilvaplėvės ertmės ir didžiosios taukinės solitarinis fibrozinis navikas, klinikinis atvejis. Buvo atlikta laparotomija. Jos metu rastas 11 cm skersmens didžiosios taukinės navikas. Navikas pašalintas atliekant radikalią omentektomiją. Pooperacinė eiga buvo normali. Kontroliniuose krūtinės ir pilvo kompiuterinės tomografijos bei dubens magnetinio branduolių rezonanso tomografijos tyrimuose, atliktuose praėjus 6 mėnesiams po operacijos, ligos atkryčio požymių nematoma.

**Išvados:**

Solitarinis fibrozinis navikas yra labai retas riboto piktybiškumo navikas, tačiau negydomas gali sukelti rimtų komplikacijų. Dėl klinikinių simptomų nebuvimo navikas dažniausiai aptinkamas atsitiktinai. Radikalus chirurginis naviko pašalinimas yra optimalus gydymo variantas.

## Introduction

Solitary fibrous tumors (SFT) are a rare soft tissue mesenchymal neoplasms that form less than 2% of all soft tissue tumors. They range from benign tumors to more malignant masses and are characterized by high fibrosity and hypervascularity. Tumors can vary in size from small to very large and can occur anywhere, however, some areas are more characteristic than others. They occur equally in men and women and can be diagnosed at all ages of adulthood, most commonly presenting at 50–60 years of age [1]. The solitary fibrous tumors are derived from the mesothelial origin, nevertheless, numerous cases of tumors of extraserosal sites are reported. SFTs are classified into pleural and extrapleural tumors. Although SFT can appear in any location of the human body, abdominal localization is quite rare [2]. Eight percent out of 79 SFT cases occurred in the peritoneal, visceral, or retroperitoneal area, and 16% in the pelvis [3]. The majority of the SFTs remain benign and asymptomatic unless they place pressure on adjacent tissues, making them hard to discover [4]. Unlike thoracic, extrathoracic solitary fibrous tumors causes symptoms such as pain or pressure, depending on the size and localization of the tumor mass [5]. In some cases STFs show malignancy and invasion of surrounding tissues [4]. Thus, it is essential to perform complete surgical removal of the tumor because of the possibility of malignant transformation and metastatic spread. Moreover, histopathology not always indicates the biological behavior of the tumor. Therefore, in some cases, SFTs can recurrent and metastasize after surgical removal. For this reason, long-term clinical follow-up is obligatory for all patients [5]. The aim of our article is to present a rare case of an asymptomatic intra-abdominal solitary fibrous tumor in a young man.

## Case report

A 32-year-old man was admitted to the abdominal surgery department due to findings on abdominal ultrasound examination. The examination was performed during preventive health check-up due to work policy. During the ultrasound examination asymptomatic suspicious mass was detected in the pelvis. Magnetic resonance imaging (MRI) with intravenous contrast was performed (Figure 1). In the pelvis, above the bladder, a mass of a heterogeneous structure with a predominant solid component and several calcinates was visible. The mass had uneven vascularization with diffusion restriction zones, had a polycyclic margin, and was about 80x107x80 mm in size. Multiple small blood vessels were seen in the peripheral part of the mass arising from the basin of the superior mesentery artery. The mass appeared to be in close contact with the upper part of the bladder and middle-distal part of the sigmoid colon with no visible infiltration into the surrounding organs. No pathological changes were found during the colonoscopy. Cystoscopy and urethroscopy showed no changes as well.

Figure 1.MRI images of the pelvis.

**a):**
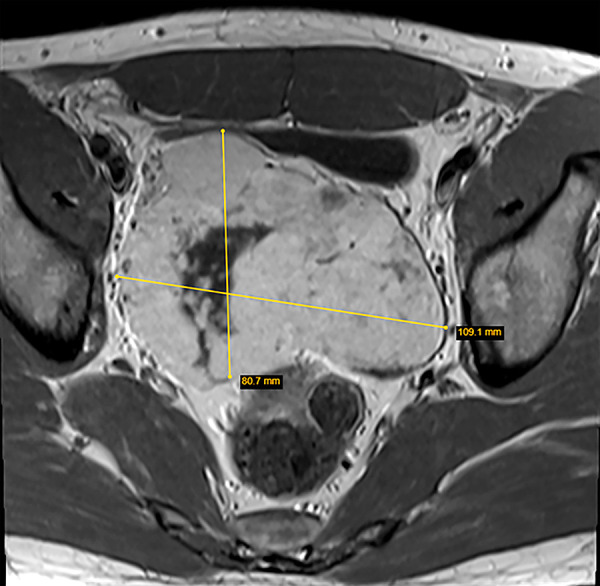
T1 transverse image of the pelvis with the tumor measured.

**b):**
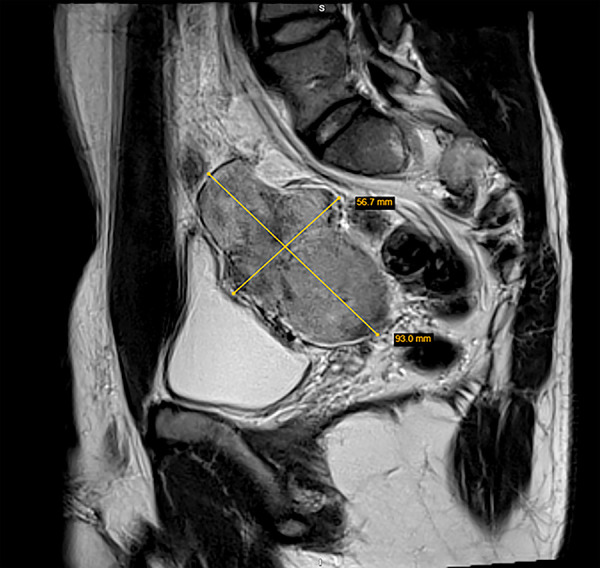
T2 sagittal image of the pelvis with the tumor measured.

**Figure 2.**
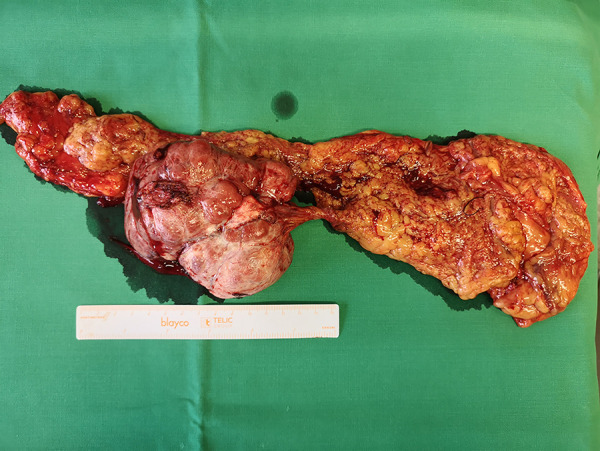
Solitary fibrous tumor and the omentum removed.

**Figure 3.**
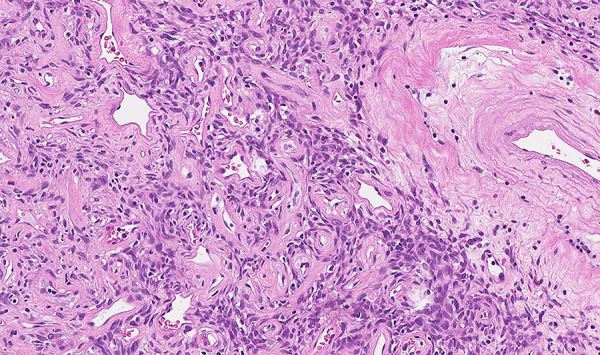
The tumor consists of spindled to ovoid cells arranged in fascicles in collagenized stroma with branching , dilated and hyalinized blood vessels.

A multidisciplinary team discussion took place, and the decision for surgery was taken. A middle-lower laparotomy was performed on a patient. An 11 cm tumor was found, beginning in the greater omentum, and it was fixed by loose vascularized adhesions to the rectum in two places and the posterior-right lateral wall of the bladder. Adhesions were excised radically and the biopsy from the areas of adhesions were taken. As the tumor was in the greater omentum and dilated veins up to 7–8 mm were going to the splenic hilum, the greater omentum was mobilized from the transverse intestine and radical omentectomy was performed (Figure 2).

**Figure 4.**
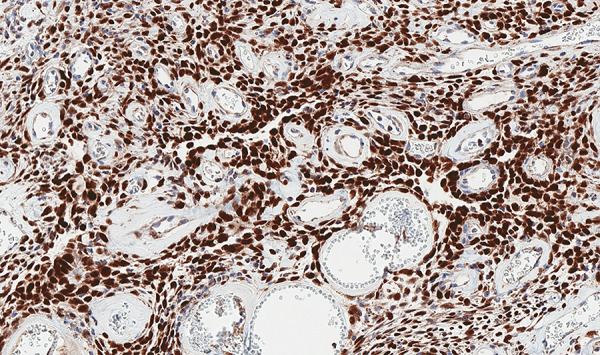
100% of tumor cells show positive nuclear STAT6 staining.

On pathological examination, macroscopically observed grayish-white, stiff, coarse-grained tumor 10.5 × 9.5 × 5.2 cm with omentum 26 × 14 × 1 cm was identified. Histologically the peritoneal tumor consisted of fibrous and fibromyxoid tissue with vessels of various sizes and uneven cellularity, irregular fibrous squamous cells with monomorphic oval/oblong nuclei, and a small number of typical mitoses (up to 3 mitoses per 10 high-powered fields (HPFs)) (Figure 3). Perivascular and stromal hyalinization were observed as well as focal tumor necrosis. No metastases into omentum lymph nodes were found. Immunohistochemical analysis revealed that the tumor was positive for STAT6 and CD34 (Figure 3 and Figure 4). The Ki-67 index was up to 5%. Histological findings support the diagnosis of the peritoneal cavity and greater omentum solitary fibrous tumor.

**Figure 5.**
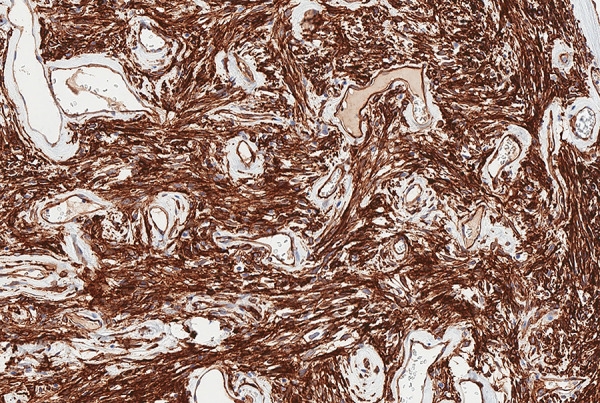
100% of tumor cells show positive cytoplasmic CD34 staining.

A postoperative course was uneventful and the patient was discharged 7 days after his surgery. The patient is well and no signs of recurrence are visible on the chest and abdomen CT and the pelvic MRI 6 months after the surgery.

## Discussion

We describe a rare case of the intra-abdominal solitary fibrous tumor in a young man. It was first described as a distinct mesothelial tumor by Klemperer and Rabin in 1931. But over time it became clear that the origin of the tumor is more mesenchymal rather mesothelial due to numerous tumor cases of other extraserosal sites. Thus, it is classified into pleural and extrapleural tumors according to their morphology [2]. However, it can appear in any anatomical location of the human body and has unpredictable behavior. Approximately 78–88% of SFTs are benign and 12–22% are characterized as malignant [6]. Local re-growth of the tumor after removal or onset of metastasis depends mostly on the prognostic parameters such as histological findings (nuclear atypia, increased cellularity, necrosis, and more than 4 mitoses per 10 high-powered fields (HPFs)), localization (about 10–15% of SFTs located not in the thoracic cavity are recurrent or metastatic), diameter (>10 cm) and resectability [2][7]. Most common place for SFT to appear is pleura with 37% of cases; intra-abdominal SFTs are very rare; peritoneal, visceral, and retroperitoneal SFTs contain only 8% of cases [3]. A literature search revealed 14 SFTs of omentum (Table 1). Intra-abdominal SFTs are usually asymptomatic until they are big enough to cause a mass effect on other organs. The size of the tumor is highly variable and it depends on the location of the body. Typically, the size of the SFT is about 7–10 cm in diameter but may vary in a range from 1 cm to 40 cm [1].

**Table 1.** M – male, F – female, NROM – no recurrence or metastasis. Case reports written in English language.

**Case No.**	**Reference**	**Age (years)/Sex**	**Scale (mm)**	**Omentum (greater/lesser)**	**CD34**	**Histological malignancy**	**Follow-up**
1	Vasdeki et al. [8]	72/M	110	Greater	+	Yes	Not reported
2	Jung et al. [9]	57/M	180	Greater	+	Yes	32 months/ NROM
3	Koto et al. [10]	41/F	300	Greater	+	No	Not reported
4	Uemura et al. [11]	40/M	110	Greater	+	No	2 years/ NROM
5	Harada et al. [12]	62/F	100	Not specified	+	Yes	48 months/ NROM
6	Archid et al. [13]	25/M	83	Greater	+	Not specified	4 years/ NROM
7	Urabe et al. [14]	52/M	16	Greater	+	No	11 months/ NROM
8	Guo et al. [15]	64/F	270	Greater	-	Yes	Died 1.5 years later due to tumor recurrence and metastasis
9	Eltawil et al. [16]	63/M	114	Greater	+	No	6 months/ NROM
10	Cazejust et al. [17]	68/F	45	Greater	+	No	Not reported
11	Ng et al. [18]	88/M	166	Lesser	+	Yes	5 months/ NROM
12	Ingle et al. [19]	37/F	60	Greater	+	Yes	Not reported
13	Zong et al. [6]	29/M	280	Greater	+	Yes	48 months/ NROM
14	Tarrega et al. [20]	34/F	60	Greater	+	Yes	32 months NROM
15	Present case	32/M	105	Greater	+	No	6 months/ NROM

The diagnosis of the extrathoracic SFT is quite complicated since the tumor usually does not cause any symptoms or have nonspecific symptoms. Mostly, it causes pain or pressure, depending on the size and surrounding tissues and organs [5]. Thus, it is usually discovered accidentally during imaging for different reasons. On ultrasound examination, the tumor is seen as a nodule with well-defined borders and a homogenous structure. The plain chest radiography may show a well-defined various size mass originating from the pleura and in some cases may have a pedicle. The computed tomography (CT) with contrast may show a well-defined, often lobulated, hypervascular mass, and if the mass is large, it will have an area of necrosis. The magnetic resonance imaging (MRI) will show an inhomogeneous well-defined mass with large areas of necrosis as bright signaling reflective extensive areas [1][2][4]. A pre-treatment biopsy of the tumor is also recommended [1].

On inspection the solitary fibrous tumor appears as yellow-tan to white, well-circumscribed, firm mass, and is often attached by a pedicle and partially encapsulated. Stromal bleeding and necrosis are also very common [2][21][22]. Histologically, characteristic diagnostic features for a benign SFT are as follows: circumscription; juxtaposed hyper- and hypocellular cell proliferation; bland-looking spindle or oval cells with scanty and poorly defined cytoplasm; up to 3 mitoses in 10 HPF; random, storiform, or fascicular arrangement of spindly cells; the close intertwining of thin or thick collagen fibrosis with spindly cells [2][14][21]. Characteristics of the SFT malignancy are 4 or more mitoses in HPF; hemorrhage; necrosis; nuclear atypia; infiltrative margins [1][2][14]. Immunohistochemistry revealed that the most consistent conventional marker of SFT is CD34 with expression in 79% of cases. Other markers such as vimentin, CD99, BCL2, nuclear β-catenin, and epithelial membrane antigen (EMA) may be expressed in SFT [1][21]. Moreover, strong STAT6 and ALDH1 expression observed using immunohistochemistry has been shown to be a highly sensitive and specific diagnostic marker for SFTs [21][23].

The treatment of the solitary fibrous tumor should be performed by a multidisciplinary medical team including surgeons, medical oncologists, radiation oncologists, and additional ancillary support. The main treatment strategy of SFT is the surgical removal of the tumor. The removal of SFT is similar to other soft tissue tumor removal with a wide resection margin that decreases the rate of tumor recurrence or metastases [5]. Radiation therapy as a treatment option is not studied enough to this day and there is a lack of data due to the rarity of this tumor. However, radiation therapy is not recommended after complete tumor resection with negative margins, but adjuvant radiation can be used with positive margins or recurrent tumors. Chemotherapy outcomes treating SFTs are also unclear due to a lack of data. Retrospective studies have shown low or questionable rates of response to chemotherapy [1][21].

Surveillance for recurrence or risk for metastases should be specific for every patient dependent on tumor localization and other patient factors. More attention requires for patients whose primary tumors were greater than 15 cm in diameter with mitotic figures greater than or equal to 4/10 HPF, and who are older than 55 [1].

## Conclusion

The solitary fibrous tumor is a rare usually benign soft tissue tumor often detected by accident due to lack of symptoms. It may appear in any location of the body, but most often it is detected in the pleura. SFTs are classified into pleural and extrapleural tumors due to their origins. The most consistent biomarkers for SFT are CD34, CD99, STAT6, and ALDH1. The main treatment option for SFT is surgery while radiotherapy treatment and chemotherapy are considerable due to lack of data.
